# Psychometric properties of the GAD-7 score to detect generalized anxiety in health professionals in Bolivia

**DOI:** 10.17843/rpmesp.2022.391.8620

**Published:** 2022-03-31

**Authors:** María F. Porto, Ninoska Ocampo-Barba, Gilda Flores-Valdivia, Nicole Caldichoury, Norman López

**Affiliations:** 1 Universidad de la Costa, Barranquilla, Colombia. Corporación Universitaria de la Costa Universidad de la Costa Barranquilla Colombia; 2 Universidad Autónoma Gabriel René Moreno, Santa Cruz de la Sierra, Bolivia. Universidad Autónoma Gabriel René Moreno Universidad Autónoma Gabriel René Moreno Santa Cruz de la Sierra Bolivia; 3 Universidad Continental, Arequipa, Peru. Universidad Continental Arequipa Peru; 4 Universidad de Los Lagos. Osorno, Chile. Universidad de Los Lagos Universidad de Los Lagos Osorno Chile

*To the editor*. The increase in cases and deaths related to COVID-19 has caused a significant physical and emotional toll on healthcare professionals. Healthcare workers have had to deal with stigma and social rejection, the risk of becoming ill, and the fear of infecting their families, which could decrease their psychological well-being and increase mental health problems ^1^. Generalized anxiety is a mental disorder in which a person is worried or anxious about one or more situations and finds it difficult to control their symptoms. Available evidence shows an increase in cases of generalized anxiety in medical personnel who are in direct contact with patients infected with COVID-19 ^2^. During the pandemic, the overall burden of anxiety in healthcare workers increased by more than 30% ^3^.

Therefore, the use of instruments that are brief, easy to apply and capable of detecting psychological disturbances in the current pandemic, has become relevant ^4^. The seven-item anxiety score (GAD-7) (Supplementary Material) was originally developed in English as a brief screening tool capable of detecting anxiety ^5^. The original study reported adequate sensitivity (0.92) and specificity (0.83) values. Since then, its psychometric properties have been examined ^6^. In Latin America, studies have been reported in Peru ^7^ and Colombia ^8^. However, there are no published data in Bolivia on the psychometric characteristics of this instrument. Therefore, we analyzed the psychometric properties of the GAD-7 score in a large sample of Bolivian health professionals during the COVID-19 pandemic.

A cross-sectional measurement was carried out from June 2020 to April 2021 on Bolivian health personnel working in hospitals and clinics, involved or not in the care of patients with COVID-19, who completed an online survey received by email or WhatsApp. We used non-probability purposive sampling. The protocol included informed consent, the GAD-7 score and four demographic questions. The GAD-7 score is a brief instrument with the following response options “not at all”, “several days”, “more than half of the days”, and “almost every day”, scored as 0, 1, 2, and 3, respectively. A score of 10 or more represents a cut-off point for identifying anxiety. The cut-off points of 5, 10 and 15 can be interpreted at mild, moderate and severe anxiety levels ^5^. We used the confirmatory factor analysis (CFA) based on a weighted least squares model with variance adjusted, considering the original structure of the score (1 factor). Finally, we conducted an internal consistency analysis through McDonald’s omega coefficient (ω). This research is part of an international multicenter study, led by the Universidad de la Costa (Act: 86-2020, research project code: INV.140-02-004-15).

The final sample consisted of 757 participants (mean age: 39.73; standard deviation: 7.88). Most of the professionals were specialists and general practitioners (48.4%), followed by nurses (27.3%), and residents (7.1%); the remaining 11.8% were clinical support professionals; 45.6% worked in private clinics (n=345) and 54.4% in the public system (n=412); 51.4% of the personnel dedicated their functions to the attention and care of patients infected with coronavirus.

Table 1 shows the descriptive values of the GAD-7 items. Skewness showed values less than 1 and kurtosis less than 2, so the distribution of the data did not affect further processing. The CFA showed an adequate fit (Root Mean Square Error of Approximation, RMSEA=0.065; Goodness of Fit Index, GFI=0.999; Adjusted Goodness of Fit Index, aGFI=0.995; Root Mean Square Residuals, SRMR=0.051) for the one-factor model. We observed factor loadings ranging from 0.68 to 0.89 (Table 1). The correlation between items is low, which does not affect discriminant validity. Finally, the internal consistency analysis showed a ω of 0.5; which demonstrates high reliability of the scale.

**Table 1:** Descriptive and factor loadings of the GAD-7 score items assessed in health professionals in Bolivia during June 2020 and April 2021.

N°	Item	Mean	SD	Kurtosis	Skewness	Factor loadings
1	In the last 15 days, have you felt nervous, anxious, on edge?	1.39	0.93	-0.79	0.24	0.86
2	In the last 15 days, have you had any inability to avoid or control worry?	1.15	0.84	-0.12	0.56	0.92
3	In the last 15 days have you presented excessive worry about different things or situations?	1.45	0.89	-0.71	0.16	0.84
4	In the last 15 days have you had difficulty relaxing?	1.51	0.90	-0.79	0.15	0.88
5	In the last 15 days have you been so restless that you can’t keep still?	1.09	0.89	-0.68	0.39	0.81
6	In the last 15 days have you been easily angered or irritable?	1.37	0.94	-0.82	0.22	0.72
7	In the last 15 days have you felt afraid, as if something bad might happen?	1.40	1.01	-1.06	0.16	0.68

SD: Standard deviation

We can conclude that the GAD-7 score shows good indicators of validity and reliability, so it can be used for the detection of generalized anxiety in health personnel working in Bolivia. We recommend expanding research on GAD-7 with measures of sensitivity and specificity in order to determine the best cut-off point for case detection. In addition, we also recommend further research on the analysis of the psychometric characteristics of the GAD-7 in the general population.
