# Supplementary material for: Psychometric properties of the GAD-7 score to detect generalized anxiety in health professionals in Bolivia
Source: Rev Peru Med Exp Salud Publica. 2022 Mar 31;39(1):115–6. doi: 10.17843/rpmesp.2022.391.8620 (PMC11397688; doi:10.17843/rpmesp.2022.391.8620)
Supplement: Supplementary material. — Available in the electronic version of the RPMESP. [file rpmesp-39-01-8620-s001.pdf]

MATERIAL SUPLEMENTARIO

Anexo 1. Escala GAD-7.

N	Preguntas	En absoluto	Varios días	Más de la mitad de los días	Casi todos los días
1	¿En los últimos 15 días ha presentado sensación de nerviosismo, de ansiedad, de tener los nervios de punta?	0	1	2	3
2	¿En los últimos 15 días ha presentado incapacidad para eludir o controlar la preocupación?	0	1	2	3
3	¿En los últimos 15 días ha presentado preocupación excesiva por diferentes cosas o situaciones?	0	1	2	3
4	¿En los últimos 15 días ha presentado dificultad para relajarse?	0	1	2	3
5	¿En los últimos 15 días ha presentado una intranquilidad de tal grado que no puede quedarse quieto?	0	1	2	3
6	¿En los últimos 15 días ha presentado facilidad para enfadarse o irritabilidad?	0	1	2	3
7	¿En los últimos 15 días ha sentido miedo, como si pudiera suceder algo malo?	0	1	2	3

Rev Peru Med Exp Salud Publica
https://doi.org/10.17843/rpmesp.2022.391.8620
