# Supplementary material for: Psychometric properties of the GAD-7 score to detect generalized anxiety in health professionals in Bolivia
Source: Rev Peru Med Exp Salud Publica. 2022 Mar 31;39(1):115–6. doi: 10.17843/rpmesp.2022.391.8620 (PMC11397688; doi:10.17843/rpmesp.2022.391.8620)
Supplement: Translation

# Translation: Propiedades psicométricas del GAD-7 para detectar ansiedad generalizada en profesionales sanitarios de Bolivia

**Authors:** María F. Porto, Ninoska Ocampo-Barba, Gilda Flores-Valdivia, Nicole Caldichoury, Norman López

**Affiliations:** 1 Universidad de la Costa, Barranquilla, Colombia.; 2 Universidad Autónoma Gabriel René Moreno, Santa Cruz de la Sierra, Bolivia.; 3 Universidad Continental, Arequipa, Perú.; 4 Universidad de Los Lagos. Osorno, Chile.

Sr. Editor. El aumento de los casos y las muertes relacionadas con la COVID-19 ha generado un importante desgaste físico y emocional en los profesionales sanitarios. El personal de salud ha tenido que enfrentarse al estigma y al rechazo social, al riesgo a enfermar y al miedo a contagiar a sus familias, lo que podría disminuir su bienestar psicológico y aumentar los problemas de salud mental^ (^ ^1^. La ansiedad generalizada es un trastorno mental en el cual una persona está preocupada o ansiosa respecto de una o varias situaciones y le parece difícil controlar sus síntomas. La evidencia disponible muestra un incremento de la ansiedad generalizada en el personal médico que está en contacto directo con los pacientes contagiados con la COVID-19^ (^ ^2^. Durante la pandemia, la carga global de ansiedad en los trabajadores de la salud se incrementó en más del 30%^ (^ ^3^. 

Por tanto, el uso de instrumentos breves, fáciles de aplicar y capaces de detectar alteraciones psicológicas en la actual pandemia, ha tomado relevancia^ (^ ^4^. La escala de ansiedad de siete ítems (GAD-7) (Material suplementario), se desarrolló originalmente en inglés como una herramienta de detección breve capaz de detectar la ansiedad^ (^ ^5^. El estudio original reportó adecuados valores de sensibilidad (0,92) y especificidad (0,83). Desde entonces, sus propiedades psicométricas se han examinado ^6^. En Latinoamérica se reportan estudios en Perú ^7^ y Colombia ^8^. Sin embargo, no existen datos publicados en Bolivia sobre las características psicométricas de este instrumento. Por lo tanto, analizamos las propiedades psicométricas del GAD-7 en una muestra amplia de profesionales sanitarios bolivianos durante la pandemia de la COVID-19. 

Se realizó una medición transversal desde junio del 2020 hasta abril del 2021 a personal de salud boliviano que laboraba en hospitales y clínicas, involucrados o no en la atención de pacientes con la COVID-19, quienes completaron un formulario en línea recibido por correo electrónico o WhatsApp. El muestreo fue de tipo no probabilístico intencional, el protocolo incluía el consentimiento informado, la escala GAD-7 y cuatro preguntas demográficas. La escala GAD-7 es un intrumento breve con opciones de respuesta «en absoluto», «varios días», «más de la mitad de los días» y «casi todos los días», puntuados como 0, 1, 2 y 3, respectivamente. Para su calificación, 10 o más representa un punto de corte para identificar ansiedad. Los puntos de corte de 5, 10 y 15 pueden interpretarse en los niveles leves, moderados y severos ^5^. Se aplicó análisis factorial confirmatorio (AFC), en base a un modelo de mínimos cuadrados ponderados, con varianza ajustada, considerando la estructura original de la escala (1 factor). Finalmente, se efectuó un análisis de consistencia interna a través del coeficiente omega (ω) de McDonald. Esta investigación es parte de un estudio multicéntrico internacional, dirigido por La Universidad de la Costa (Acta: 86-2020, código del proyecto de investigación: INV.140-02-004-15). 

La muestra final quedó conformada por 757 participantes (promedio de edad 39,73; desviación estándar 7,88). La mayoría de los profesionales fueron médicos especialistas y generales (48,4%), seguido por enfermeras (27,3%), y residentes (7,1%); el restante 11,8%, fueron profesionales de apoyo clínico, el 45,6% laboraba en clínicas privadas (n=345) y el 54,4% en el sistema público (n=412); el 51,4% del personal dedicó sus funciones a la atención y cuidado de pacientes contagiados por coronavirus.

La Tabla 1 muestra los descriptivos de los items del GAD-7. La asimetría mostró valores menores a 1 y la curtosis menores a 2, por lo que la distribución de los datos no afectó el procesamiento posterior. El AFC mostró un ajuste adecuado (Raíz cuadrada media del error de aproximación, RMSEA=0,065; Índice de Bondad de ajuste, GFI=0,999; Índice ajustado de calidad de ajuste, aGFI=0,995; Raíz cuadrada de la media de residuos, SRMR=0,051) para el modelo de un factor. Observamos cargas factoriales que oscilan de 0.68 a 0.89 (Tabla 1). Por último, la correlación entre los ítems es baja, lo cual no afecta la validez discriminante. Finalmente, el análisis de consistencia interna mostró un ω de 0,5; lo cual muestra una alta fiabilidad de la escala.

**Tabla 1:** Descriptivo y cargas factoriales de los ítems de la escala GAD-7 evaluado en profesionales sanitarios de Bolivia durante junio 2020 y abril de 2021.

Nº	Ítem	Media	DE	Curtosis	Asimetría	Cargas factoriales
1	¿En los últimos 15 días ha presentado sensación de nerviosismo, de ansiedad, de tener los nervios de punta?	1,39	0,93	-0,79	0,24	0,86
2	¿En los últimos 15 días ha presentado incapacidad para eludir o controlar la preocupación?	1,15	0,84	-0,12	0,56	0,92
3	¿En los últimos 15 días ha presentado preocupación excesiva por diferentes cosas o situaciones?	1,45	0,89	-0,71	0,16	0,84
4	¿En los últimos 15 días ha presentado dificultad para relajarse?	1,51	0,90	-0,79	0,15	0,88
5	¿En los últimos 15 días ha presentado una intranquilidad de tal grado que no puede quedarse quieto?	1,09	0,89	-0,68	0,39	0,81
6	¿En los últimos 15 días ha presentado facilidad para enfadarse o irritabilidad?	1,37	0,94	-0,82	0,22	0,72
7	¿En los últimos 15 días ha sentido miedo, como si pudiera suceder algo malo?	1,40	1,01	-1,06	0,16	0,68

DE: desviación estándar

Podemos concluir que el GAD-7 muestra buenos indicadores de validez y confiabilidad, por lo que puede ser utilizado para la detección de ansiedad generalizada en personal sanitario que trabaja en Bolivia. Recomendamos ampliar su estudio con medidas de sensibilidad y especificidad para determinar el mejor punto de corte para la detección de casos. Además, expandir los estudios que analicen las características psicométricas del GAD-7 en población general.
